# Factors related to mean corpuscular volume in *HFE* p.C282Y homozygotes

**DOI:** 10.1002/jha2.1063

**Published:** 2024-12-21

**Authors:** James C. Barton, J. Clayborn Barton, Ronald T. Acton

**Affiliations:** ^1^ Department of Medicine University of Alabama at Birmingham Birmingham Alabama USA; ^2^ Southern Iron Disorders Center Birmingham Alabama USA; ^3^ Department of Microbiology University of Alabama at Birmingham Birmingham Alabama USA

**Keywords:** alcohol consumption, erythrocyte, hemochromatosis, iron, macrocytosis, transferrin saturation

## Abstract

**Introduction:**

The aim of this study was to define the relationships between factors other than transferrin saturation (TS) to mean corpuscular volume (MCV) and macrocytosis (MCV > 100 fL) in *HFE* p.C282Y (rs1800562) homozygotes.

**Methods:**

We studied white post‐screening participants with p.C282Y homozygosity who did not have anemia, report cirrhosis or pregnancy, or use medications that increase MCV. We analyzed relations of MCV and macrocytosis with age, sex, diabetes reports, daily alcohol consumption, swollen or tender 2nd/3rd metacarpophalangeal (MCP) joints, TS, and serum ferritin (SF).

**Results:**

There were 257 participants (110 men and 147 women). Median alcohol consumption, median TS, median SF, and macrocytosis prevalence were significantly greater in men. Relative risk of macrocytosis in men was 2.81. In men and women, there were significant positive Pearson's correlations of MCV versus age and Spearman's correlations of MCV versus alcohol consumption and TS. Mean MCV and macrocytosis prevalence were significantly greater in participants with than without swollen or tender 2nd/3rd MCP joints. Linear regression on MCV revealed positive associations: age (*p* < 0.0001), alcohol consumption (*p* = 0.0007), and TS (*p* < 0.0001). Logistic regression on macrocytosis revealed an odds ratio for age of 1.04 (95% confidence interval: 1.00, 1.07).

**Conclusions:**

MCV in *HFE* p.C282Y homozygotes is positively related to age, daily alcohol consumption, TS, and swollen or tender 2nd/3rd MCP joints.

## 1 INTRODUCTION

Hemochromatosis in white people of European descent is usually associated with homozygosity for p.C282Y (rs1800562), a missense allele of the homeostatic iron regulator (*HFE*, chromosome 6p22.2) [1, 2]. HFE, a nonclassical class I major histocompatibility complex protein, is an upstream regulator of hepcidin and thus of iron homeostasis [3]. At diagnosis of p.C282Y homozygosity, many adults have elevated transferrin saturation (TS) [4] caused by increased recycling of iron from senescent erythrocytes and iron stores [5]. At diagnosis of p.C282Y homozygosity, some adults also have elevated serum ferritin (SF) [4], a surrogate marker of iron overload [6]. In studies of referred patients with p.C282Y homozygosity, 14.5% had diabetes [7] and 55.5% had hemochromatosis‐associated arthropathy [8].

Mean corpuscular volume (MCV), the average volume of erythrocytes [9], is significantly greater in *HFE* p.C282Y homozygotes at diagnosis than control subjects [10, 11, 12, 13, 14]. MCV in p.C282Y homozygotes was positively related to TS and SF in three studies [10, 13, 14]. It was postulated that the increased mean MCV in adults with hemochromatosis is due primarily to the increased uptake of transferrin‐bound iron by immature erythroid cells and consequent increased hemoglobin (Hb) synthesis [10]. Although four subsequent genome‐wide association studies (GWAS) revealed positive statistical associations between MCV and p.C282Y [15, 16, 17, 18], it is unlikely that *HFE* alleles directly alter erythroid iron handling or Hb synthesis because HFE protein is not expressed in erythroid colonies with a normal *HFE* genotype [19]. It is plausible that factors other than TS and SF either influence or are related to MCV in p.C282Y homozygotes.

MCV increased with age in English adults in primary care venues [20]. The mean MCV in Scottish adults with diabetes was significantly higher than that of control subjects [21]. MCV in white British adults without anemia increased linearly with alcohol intake [22]. We found no description of the relationships of MCV or macrocytosis with age, reports of diabetes, or daily alcohol consumption in HFE p.C282Y homozygotes who did not have anemia, report cirrhosis or pregnancy, or use medications that increase MCV.

The aim of this study was to define the relationships between post‐screening MCV and macrocytosis and age, sex, reports of diabetes, daily alcohol consumption, swollen or tender 2nd/3rd metacarpophalangeal (MCP) joints, TS, and SF in 257 non‐Hispanic white *HFE* p.C282Y homozygotes who participated in the Hemochromatosis and Iron Overload Screening (HEIRS) Study. We compare and discuss our observations with those in previously reported population cohorts, and propose the potential clinical relevance of our results.

## 2 METHODS

### 2.1 Participants

The HEIRS Study primary care‐based screening is described in detail elsewhere [4, 23]. Homozygosity for *HFE* p.C282Y was identified in 299 HEIRS Study participants [23]. Invitations to attend post‐screening evaluations were extended to all participants with p.C282Y homozygosity. Attendees included 287 non‐Hispanic white participants with p.C282Y homozygosity (122 men and 165 women). We excluded those who had anemia, reported cirrhosis or pregnancy, or used medications that increase MCV [24], and those whose data were incomplete. The proportions of excluded men and women did not differ significantly (9.0% vs. 10.3%, respectively; *p* = 0.8412). There were evaluable observations on 257 participants (110 men and 147 women).

### 2.2 Evaluations

Post‐screening evaluations included the following: (1) questionnaires completed by participants that addressed medical histories and medications; (2) University of Hawaii Multiethnic Dietary Questionnaires completed by participants to permit estimates of their average daily consumption of alcohol during the preceding year [25, 26]; (3) focused physical examinations performed by HEIRS Study physicians [27]; and (4) laboratory testing of blood specimens [27]. The median interval between primary care‐based screening and post‐screening evaluations was 8 months [25, 27].

### 2.3 Questionnaires and physical examinations

Participant reports of diabetes were defined as affirmative responses to the question: “Have you ever been told you have diabetes?” We compiled antihyperglycemic medications reported by participants with diabetes reports. Responses to multiethnic dietary questionnaires were analyzed at the University of Hawaii [26]. Alcohol consumption was expressed as g/d. Physicians recorded the presence or absence of swelling or tenderness of the 2nd/3rd MCP joints [27].

### 2.4 Laboratory testing

A blood sample was obtained from each participant after an overnight fast of ≥8 h [28]. Testing was performed at the HEIRS Study central laboratory (Fairview‐University Medical Center Clinical Laboratory, University of Minnesota) [27]. Reference ranges for TS and SF in men were 10%–55% and 30%–300 µg/L, respectively, and in women were 10%–45% and 20%–200 µg/L, respectively. We defined anemia as Hb < 130 g/L (men) and < 120 g/L (women). We defined the reference range of MCV as 80.0–100.0 fL. We defined macrocytosis and microcytosis as MCV > 100.0 fL and MCV < 80.0 fL, respectively.

### 2.5 Statistics

The dataset for analysis consisted of observations on 257 non‐Hispanic white participants with *HFE* p.C282Y homozygosity [29]. Kolmogorov–Smirnov testing demonstrated that age, Hb, and MCV data did not differ significantly from those that are normally distributed. Thus, we displayed these data as means ± 1 standard deviation and compared means using the Student's *t*‐test for unpaired samples (two‐tailed). Daily alcohol consumption, TS, SF, and RDW data differed significantly from those that are normally distributed. Thus, we displayed these data as medians (ranges) and compared medians using the Mann–Whitney *U* test (two‐tailed). We compared categorical data using Fisher's exact test (two‐tailed).

We computed correlations of MCV versus age, alcohol consumption, and TS in men and women using Pearson's correlation coefficient (*r*) or Spearman's rank correlation coefficient (*r_s_*) for unpaired data (two‐tailed), as appropriate, and rated the strengths of the coefficients [30]. We compared the correlations of MCV versus age in men and women using sample sizes, slopes, and standard errors. We computed differences between correlation coefficients using z‐score analyses.

We performed linear regression on MCV. After preliminary analyses, we eliminated some factors to minimize collinearity. In a final regression, we used these six independent variables: age, sex, diabetes reports, daily alcohol consumption, swollen or tender 2nd/3rd MCP joints, and TS.

We performed regression on macrocytosis. After preliminary analyses, we eliminated some factors to minimize collinearity and maximize pertinence. In univariable comparisons of participants with and without macrocytosis, we observed values of *p* < 0.2000 in the remaining six independent variables: age, sex, daily alcohol consumption, swollen or tender 2nd/3rd MCP joints, TS, and SF. We expressed some results as odds ratios (95% confidence interval (CI)).

We used Excel^®^ 2000 (Microsoft Corp.) and GraphPad Prism 8^®^ (2018; GraphPad Software). We defined *p* < 0.05 to be significant.

## 3 RESULTS

### 3.1 Characteristics of 257 *HFE* p.C282Y homozygotes

There were 110 men (42.8%) and 147 women (57.2%). The mean ages of men and women did not differ significantly (Table 1). Median daily alcohol consumption, median TS, median SF, and mean Hb were significantly greater in men than women (Table 1). The mean MCV of men and women did not differ significantly (Table 1), although the mean MCV of men aged ≥50 years was greater than that of women aged ≥50 years (97.0 ± 5.2 fL vs. 95.4 ± 4.3 fL, respectively; *p* = 0.0419).

**TABLE 1:** *HFE* p.C282Y homozygotes in a post‐screening evaluation.

Characteristic	Men (*n* = 110)	Women (*n* = 147)	*p* value
Mean age, y ± 1 SD	53 ± 14	51 ± 13	0.4654
Diabetes reports, % (*n*)	13.6 (15)	7.5 (11)	0.1426
Median alcohol consumption, g/d (range)	3.1 (0, 81.7)	1.2 (0, 107.3)	0.0276
Swollen or tender 2nd/3rd MCP joints, % (*n*)	17.3 (19)	12.2 (18)	0.2840
Median TS, % (range)	80 (20, 100)	61 (10, 100)	0.0012
Median SF, µg/L (range)	580 (24, 5300)	237 (8, 5060)	<0.0001
Mean Hb, g/L ± 1 SD	154 ± 9	140 ± 9	<0.0001
Mean MCV, fL ± 1 SD	95.8 ± 5.1	94.8 ± 4.4	0.1055
Median RDW, % (range)	12.6 (11.0, 16.0)	12.4 (11.0, 15.4)	0.0555

*Note*: Among the 26 participants with diabetes reports, 20 (76.9%) reported that they took one or more antihyperglycemic medications.

Abbreviations: Hb, hemoglobin; MCP, metacarpophalangeal; MCV, mean corpuscular volume; RDW, red blood cell distribution width; SD, standard deviation; SF, serum ferritin; TS, transferrin saturation.

The prevalence of diabetes reports was 10.1%. The mean MCV of participants with and without diabetes reports did not differ significantly (94.8 ± 4.1 fL vs. 95.2 ± 4.8 fL, respectively; *p* = 0.6289). The prevalence of swollen or tender 2nd/3rd MCP joints was 14.4%. The mean MCV was greater in participants with than without swollen or tender 2nd/3rd MCP joints (97.1 ± 5.1 fL vs. 94.9 ± 4.6 fL, respectively; *p* = 0.0192).

### 3.2 Relationships of MCV and age

There was a positive Pearson's correlation of MCV versus age in men (*r*_110_ = 0.3608; *p* = 0.0001), although the strength of this correlation was fair (Figure S1). There was a positive Pearson's correlation of MCV versus age in women (*r*_147_ = 0.2262; *p* = 0.0059), although the strength of this correlation was poor (Figure S2).

The slope of the regression line of MCV versus age was greater in men (0.1362) than women (0.0776), although the difference was not significant (*t* = 0.1754; *p* = 0.8609). The corresponding correlation coefficients did not differ significantly (z‐score = 1.1566; *p* = 0.2474).

### 3.3 Correlations of MCV versus alcohol consumption and transferrin saturation

There were significant positive Spearman's rank correlations of MCV versus daily alcohol consumption in men and women (*p* = 0.0018 and *p* = 0.0015, respectively) (Figures S3 and S4). There were significant positive Spearman's rank correlations of MCV versus TS in men and women (*p* = 0.0018 and *p* = 0.0012, respectively) (Figures S5 and S6).

### 3.4 Regression on MCV

Linear regression on MCV revealed three positive associations: age (*p* < 0.0001), alcohol consumption (*p* = 0.0007), and TS (*p* < 0.0001). This regression accounted for 19.5% of the variance in MCV (ANOVA *p* of regression < 0.0001).

### 3.5 Factors affecting macrocytosis

We observed significant positive associations of macrocytosis with age, male sex, swollen or tender 2nd/3rd MCP joints, and SF in univariable comparisons (Table 2). The relative risk of macrocytosis in men was 2.81. The relative risk of swollen or tender 2nd/3rd MCP joints in participants with macrocytosis was 2.27. No participant had microcytosis.

**TABLE 2:** Comparison of *HFE* p.C282Y homozygotes with and without macrocytosis.

Characteristic	Macrocytosis (*n* = 31)	No macrocytosis (*n* = 226)	*p* value
Mean age, y ± 1 SD	59 ± 12	51 ± 13	0.0021
Men, % (*n*)	67.7 (21)	39.4 (89)	0.0035
Diabetes reports, % (*n*)	9.7 (3)	10.2 (23)	∼1.0000
Median alcohol consumption, g/d (range)	7.0 (0.001, 79.7)	1.6 (0, 107.3)	0.0930
Swollen or tender 2nd/3rd MCP joints, % (*n*)	29.0 (9)	12.4 (28)	0.0320
Median TS, % (range)	81 (20, 99)	67 (10, 100)	0.1499
Median SF, µg/L (range)	591 (35, 5300)	368 (8, 3717)	0.0308

*Note*: We defined macrocytosis as MCV > 100 fL.

Abbreviations: MCP, metacarpophalangeal; MCV, mean corpuscular volume; RDW, red blood cell distribution width; SD, standard deviation; SF, serum ferritin; TS, transferrin saturation.

### 3.6 Regression on macrocytosis

Logistic regression on macrocytosis revealed two positive associations: age (*p* = 0.0292) and SF (*p* = 0.0303). This regression accounted for 13.6% of the deviance of macrocytosis (*p* of regression = 0.0003). Odds ratio of age was 1.04 (95% CI: 1.00, 1.07). Odds ratio of SF was 1.00 (95% CI: 1.00, 1.00).

## 4 DISCUSSION

Novel findings of this study of *HFE* p.C282Y homozygotes include significant positive correlations of MCV with age and daily alcohol consumption and the greater mean MCV and prevalence of macrocytosis among participants with swollen or tender 2nd/3rd MCP joints.

In this study, MCV was significantly related to age, after adjustment for other variables. This corresponds to previous reports of significant positive correlations between MCV and age in adults not selected for *HFE* p.C282Y homozygosity [20, 31]. The mean MCV was significantly higher in the present men aged ≥50 years than in women aged ≥50 years, consistent with previous observations [32]. Nonetheless, MCV was not significantly related to sex in the present study, after adjustment for other variables. Four GWAS of white persons of European descent did not identify a locus on either X or Y chromosome that was associated with MCV [15, 16, 17, 18].

Leukocyte telomere length (LTL) shortens with age [33]. In two large population studies, LTL was inversely associated with MCV [34, 35]. Thus, an effect of aging on telomere length may contribute to the significant positive association between MCV and age in the present *HFE* p.C282Y homozygotes. In 474,074 participants in the UK Biobank, men had significantly shorter LTL than women [36]. Thus, the shorter LTL in men than women in general populations [36] could explain in part the significantly greater mean MCV of men than women with p.C282Y homozygosity aged ≥50 years we observed in the present study.

Elevated MCV is common in persons with chronic alcoholism [37]. MCV of the present *HFE* p.C282Y homozygotes was significantly related to the quantity of daily alcohol consumption, after adjustment for age, sex, and TS. The mean MCV in Australian men and women with any p.C282Y genotype increased progressively in those who reported that they never drank alcohol, were light drinkers, or were moderate/heavy drinkers, respectively, after adjustment for age [38]. There was no evidence that the effect of alcohol consumption on MCV was different for those with and without *HFE* polymorphisms [38]. In a population study of British adults without anemia, MCV increased linearly with alcohol intake [22].

MCV was significantly related to TS in the present study. These results agree with those of another study of HEIRS Study post‐screening participants [13] and two studies of referred *HFE* p.C282Y homozygotes [10, 14]. These results are also consistent with the initial postulate that the increased mean MCV in adults with hemochromatosis is caused in part by the uptake of increased quantities of transferrin‐bound iron by immature erythroid cells and consequent increased Hb synthesis [10] and a subsequent postulate that HFE protein influences the availability of iron to erythroid cells [19].

Elevated TS and SF were associated with shorter LTL in *HFE* p.C282Y homozygotes in two studies [39, 40]. In a general Korean population, TS was inversely associated with LTL, after adjustment for other variables [41]. In a representative US population, especially in adults aged ≥65 years, high SF levels were inversely associated with LTL [42]. Thus, higher TS or SF are associated with shorter LTL, a possible consequence of iron‐induced oxidative stress on telomere length [43].

The mean percentage of reticulocytes in *HFE* p.C282Y homozygotes was 25% greater than the upper reference limit in one study [19]. The volume of individual blood reticulocytes (R2) is 100–120 fL [44]. Thus, reticulocytes may contribute to the higher mean MCV of p.C282Y homozygotes than control subjects, although we found no report that corroborates this possibility.

Mean MCV and macrocytosis in the present participants were not related to diabetes reports. In Scottish adults with diabetes, the mean MCV was significantly higher than that of control subjects, although there was no relationship between mean MCV and diabetes subtype, method of treatment, or glycemic control [21].

Participants who reported having cirrhosis or taking medications that increased MCV were excluded from the present study. Mean MCV in adults with *HFE* p.C282Y homozygosity is higher in those with than without cirrhosis [45]. Observations on MCV in adults with or without cirrhosis unselected for p.C282Y homozygosity [46] are similar to those in p.C282Y homozygotes [45]. Certain medications increase MCV in adults unselected for p.C282Y homozygosity [47]. It is assumed that the same medications have similar effects in p.C282Y homozygotes.

The mean MCV and the prevalence of macrocytosis were significantly greater among the present participants with than without swollen or tender 2nd/3rd MCP joints. In referred Australian p.C282Y homozygotes, the mean MCV was also significantly greater in those with than without 2nd/3rd MCP and other arthropathy [8]. It was postulated that the availability of iron to cells involved in the pathogenesis of hemochromatosis‐related arthropathy is increased in p.C282Y homozygotes with higher values of MCV [8].

We excluded participants who reported that they had been diagnosed to have cirrhosis. In 158 Australian population screening program participants with *HFE* p.C282Y homozygosity (74 men and 84 women), the prevalence of fibrosis or cirrhosis was low (7.6%) [48]. Thus, the absence of liver biopsy or elastography data in the present participants is unlikely to influence the present results significantly. Measuring erythrocyte volume subsets, erythrocyte survival times, erythrocyte iron uptake, serum levels of vitamin B12 and folate, reticulocyte counts, and LTLs and evaluating liver histology using biopsies and elastography were beyond the scope of the HEIRS Study. Factors other than those we studied probably also influence MCV or the occurrence of macrocytosis in p.C282Y homozygotes, including loci on chromosomes other than chromosome 6p [15].

## 5 CONCLUSION

We conclude that MCV in *HFE* p.C282Y homozygotes is positively related to age, daily alcohol consumption, TS, and swollen or tender 2nd/3rd MCP joints.

## AUTHOR CONTRIBUTIONS

Each author contributed equally to this study. James C. Barton conceived this study and its methodology, evaluated participants in post‐screening evaluations, performed analyses, and drafted the manuscript. J. Clayborn Barton conceived study methodology, curated data, performed analyses, and drafted the manuscript. Ronald T. Acton conceived this study and its methodology, performed analyses, curated data, and drafted the manuscript. Each author approved the manuscript in its final form.

## CONFLICT OF INTEREST STATEMENT

The following individuals performed data collection (2001–2003) and received compensation for their work: Paul C. Adams, MD (Department of Medicine, London Health Sciences Centre, London, Ontario, Canada); John H. Eckfeldt, MD, PhD (Department of Laboratory Medicine and Pathology, University of Minnesota, Minneapolis); Victor R. Gordeuk, MD (Division of Hematology and Oncology, Department of Medicine, University of Illinois at Chicago, Chicago); Emily Harris, PhD (Epidemiology and Genomics Research Program, Division of Cancer Control and Population Sciences, National Cancer Institute, National Institutes of Health, Bethesda, Maryland); Helen Harrison, RN (The Western‐Fanshawe Collaborative BScN Program, Fanshawe College, London, Ontario, Canada); Christine E. McLaren, PhD (Department of Epidemiology, University of California, Irvine, California); and Gordon D. McLaren, MD (Division of Hematology/Oncology, Department of Medicine, University of California, Irvine, and Department of Veterans Affairs Long Beach Healthcare System, Long Beach, California). The authors declare no conflicts of interest.

## ETHICS STATEMENT

The HEIRS Study, conducted by the National Heart, Lung, and Blood Institute/National Human Genome Research Institute, in accordance with principles of the Declaration of Helsinki, evaluated diverse aspects of hemochromatosis and iron overload in a primary care‐based sample of 101,168 adults enrolled during the interval 2001–2003 at four Field Centers in the United States and one in Canada [23]. Local Institutional Review Boards of the HEIRS Study Coordinating Center, the HEIRS Study Central Laboratory, and each Field Center approved the HEIRS Study protocol that is described in detail elsewhere [25].

## PATIENT CONSENT STATEMENT

Participants ≥25 years of age were recruited from outpatient facilities affiliated with five Field Centers and gave written informed consent for initial screening and post‐screening evaluation [23, 25].

## PERMISSION TO REPRODUCE MATERIAL FROM OTHER SOURCES

None.

## CLINICAL TRIAL REGISTRATION

The authors have confirmed clinical trial registration is not needed for this submission.

## Supporting information



Supporting Information

## Data Availability

The National Heart, Lung, and Blood Institute provides controlled access to individual participant data through the Biologic Specimen and Data Repository Information Coordinating Center (BioLINCC) (https://biolincc.nhlbi.nih.gov/studies/heirs/). Data access requires registration, evidence of local institutional review board approval or certification of exemption from institutional review board review, and completion of a data use agreement. The National Heart, Lung, and Blood Institute does not permit investigators to submit data directly to journals, related repositories, or other sources. Parties interested in obtaining the data analyzed in the present study are referred to BioLINCC.
