# Supplementary material for: Factors related to mean corpuscular volume in HFE p.C282Y homozygotes
Source: EJHaem. 2024 Dec 21;6(1):e1063. doi: 10.1002/jha2.1063 (PMC11756985; doi:10.1002/jha2.1063)
Supplement: Supplementary file 1 — Supporting Information [file JHA2-6-e1063-s001.docx]

**Supplemental Figures 1-6**

**Factors related to mean corpuscular volume in *HFE* p.C282Y homozygotes**

Running title: MCV in *HFE* p.C282Y homozygotes

James C. Barton^1,2^, J. Clayborn Barton^2^, and Ronald T. Acton^2,3^

^1^ Department of Medicine, University of Alabama at Birmingham, Birmingham, Alabama, USA

^2^ Southern Iron Disorders Center, Birmingham, Alabama, USA

^3^ Department of Microbiology, University of Alabama at Birmingham, Birmingham, Alabama, USA

James C. Barton (corresponding author): bartonjames336@gmail.com; ORCID 0000-0003-2876-8276

J. Clayborn Barton: jbarton205@gmail.com; ORCID 0000-0002-2646-4466

## Ronald T. Acton: rtakma@bellsouth.net; ORCID 0000-0002-8586-6887

**Supplemental Figure 1.** Pearson's correlation of mean corpuscular volume (MCV) vs. age in 110 men with *HFE* p.C282Y homozygosity (r_110_ = 0.3608; p = 0.0001).

**Supplemental Figure 2.** Pearson's correlation of mean corpuscular volume (MCV) vs. age in 147 women with *HFE* p.C282Y homozygosity (r_147_ = 0.2262; p = 0.0059).

**Supplemental Figure 3.** Spearman's rank correlation of mean corpuscular volume vs. alcohol consumption in 110 men with *HFE* p.C282Y homozygosity (*r*_110_ = 0.2072; p = 0.0018).

**Supplemental Figure 4.** Spearman's rank correlation of mean corpuscular volume vs. alcohol consumption in 147 women with *HFE* p.C282Y homozygosity (*r*_147_ = 0.2987; p = 0.0015).

**Supplemental Figure 5.** Spearman's rank correlation of mean corpuscular volume vs. transferrin saturation in 110 men with *HFE* p.C282Y homozygosity (*r*_110_ = 0.2944; p = 0.0018).

**Supplemental Figure 6.** Spearman's rank correlation of mean corpuscular volume vs. transferrin saturation in 147 women with *HFE* p.C282Y homozygosity (*r*_147_ = 0.2645; p = 0.0012).
